# Association between environmental heat stress and 30:15 intermittent fitness test performance in youth football players: a two-season observational study

**DOI:** 10.3389/fspor.2026.1896010

**Published:** 2026-09-29

**Authors:** Vincenzo Rago, Edgar Schwarz, Júlio A. Costa, George P. Nassis, Pedro Figueiredo

**Affiliations:** 1 College of Sport Science, University of Kalba, Sharjah, United Arab Emirates; 2 Institute of Sport and Preventive Medicine, Saarland University, Saarbrücken, Germany; 3 School of Sport, Exercise and Rehabilitation, Faculty of Health, University of Technology Sydney, Ultimo, NSW, Australia; 4 FPF Academy, Portuguese Football Federation, Oeiras, Portugal; 5 Physical Education Department, College of Education, United Arab Emirates University, Al Ain, United Arab Emirates

**Keywords:** aerobic fitness, anthropometric measurements, countermovement jump, team sports, performance, testing, wet-bulb globe temperature

## Abstract

Intermittent field tests in football are commonly conducted outdoors, exposing players to environmental heat stress. This study examined the influence of environmental heat stress on the 30:15 Intermittent Fitness Test (V_IFT_) performance in highly trained young football players. A total of 642 V_IFT_ assessments were collected from 312 male players (U14–U19) across two seasons. Environmental conditions were obtained from an open-source service. Heat stress was quantified via the wet-bulb globe temperature (WBGT) and categorized into unrestricted (<22 °C), low-risk (22 °C–28 °C) and high-risk (>28 °C) conditions. Linear mixed models were used to analyze the associations between anthropometric, neuromuscular, and environmental factors on V_IFT_ performance. V_IFT_ decreased by ∼0.4 km/h in low-risk and ∼1.5 km/h in high-risk heat conditions (*d* = 0.12–0.45; *P* < 0.01) compared to unrestricted. These differences persisted after adjusting for anthropometric characteristics and CMJ performance, except in U19 players, where the V_IFT_ decrement in low-risk conditions disappeared (*P* = 0.183). The V_IFT_ declined by 0.09–0.15 km/h for every 1 °C increase in WBGT (*r* = −0.74 to −0.28; *P* < 0.05). Our findings suggest the V_IFT_ performance might decrease by one running stage (0.5 km/h) for every ∼5 °C increase in WBGT. Practitioners should account for environmental conditions when profiling players and prescribing training based on the V_IFT_.

## 1 Introduction

Young football (soccer) players (aged 12–19 years) need a combination of physical capacities stemming from strength, power, speed and endurance to be successful (1). Specifically, intermittent exercise capacity is considered a key performance indicator based on its strong correlation with match physical outputs in young football players (2, 3). As intermittent field tests in football are typically conducted outdoors, there is growing concern around their repeatability and sensitivity (4, 5). This concern is based on the solid knowledge that heat stress can affect endurance performance in different sports (6), including football (5, 7, 8). This is particularly important in youth athletes, who are more susceptible to heat stress due to their immature thermoregulatory systems, including lower sweat rates, greater surface-area-to-mass ratio, and less effective cardiovascular responses, leading to decreased heat tolerance compared to adults (6, 9).

Depending on the geographical areas, young football players compete across a wide range of environmental conditions, with match temperatures reported from ∼9.2 °C in Under-14 boys from the UK (10) to ∼36 °C with 25% relative humidity in Under-17 players in Australia (11). Playing football in the heat imposes substantial physiological strain, as the combination of exercise-induced metabolic heat production and high ambient temperatures challenges the body's ability to maintain thermal homeostasis (6). To quantify this environmental heat stress, air temperature alone is insufficient, as it does not account for the combined effects of humidity, wind speed, and solar radiation on the body. The wet-bulb globe temperature (WBGT) integrates these factors into a single composite index and has been adopted as the standard tool for assessing heat stress in football (7, 8) among other Olympic sports (12, 13). Although direct on-site measurement represents the gold standard, WBGT can be reliably estimated from meteorological data available through open-source services (14).

Compared to match-derived physical outputs, standardized physical tests offer a more controlled approach by eliminating match-related confounders (e.g., opponent quality, tactical decisions) thereby allowing the effects of environmental conditions on performance to be more directly attributed to heat stress. According to a recent survey, the most adopted field-based intermittent endurance protocol among practitioners involved with testing football players is the 30:15 intermittent fitness test (30:15_IFT_) (15). This is probably due to the fact that the final outcome of 30:15_IFT_ (final velocity; *V_IFT_*) can be confidently adopted based on its reliability (16) and sensitivity to training (17) to measure a combination of relevant capacities, namely maximal cardiorespiratory function, anaerobic capacity (18), neuromuscular qualities (19), and inter-effort recovery abilities (20). However, testing in the heat might introduce a meaningful bias into test outcomes, thereby affecting player profiling and training prescriptions. Therefore, estimates of the association between environmental conditions might be applied to the test score to profile players and calibrate running conditioning.

Beyond the influence of environmental conditions, it is important to consider individual characteristics that may affect performance in intermittent field tests. Indeed, intermittent exercise is associated with a combination of combination of primarily endurance (based on a moderate to large correlation with laboratory-based maximal oxygen uptake) (21) and to a lesser extent, strength and power capacities (based on a small to moderate correlation with strength and power tests) (19, 22). A recent study revealed that taller and heavier young football players presented a greater V_IFT_ performance (*r* = 0.59–0.88), whilst no relation was found with body fat (22). In the same study, players who scored higher in the countermovement jump (CMJ) test reported greater V_IFT_ performance (22). Taking together, those findings suggest that taller, heavier and more powerful players may perform better on intermittent endurance tests. However, inferring a superior heat tolerance in youth populations should be approached with caution as heat dissipation and tolerance are influenced by several interrelated factors, including body surface area-to-mass ratio, lean mass, fat mass, and metabolic heat production (6) which are not fully captured by gross anthropometric or power-related metrics. Therefore, while physical stature and power may support intermittent test performance, it remains uncertain whether these characteristics offer a physiological advantage under heat stress. This could be of importance as children traditionally displayed less effective thermoregulatory capacity and inferior thermal tolerance during exercise under heat stress compared to adults (6).

In summary, the influence of environmental heat stress on intermittent running performance has not been examined in young football players. In this context, practitioners might benefit from estimates between environmental conditions and test outcomes, which could inform adjustments to V_IFT_ results when testing is conducted under heat stress. Thus, the study aimed to examine the influence of environmental heat stress on the 30:15_IFT_ performance in young football players and to identify individual characteristics associated with heat-induced performance decrements.

## 2 Materials and methods

### 2.1 Design

Fitness tests data were collected as part of routine team monitoring across 2022/23 and 2023/24 seasons at a football academy in the Middle-East. The last running velocity of 30:15_IFT_ (V_IFT_) was the outcome variable, environmental heat conditions were the independent variables, and individual characteristics (age, height, body mass, percentage of body fat and CMJ height) were the covariates. The V_IFT_ performance in the heat was compared to a control (unrestricted) condition based on a WBGT-based heat stress classification. Then, the association between continuous WBGT and V_IFT_ was computed to generate an V_IFT_ estimate based on 1 °C increase in WBGT. Individual characteristics were entered as covariates to examine whether anthropometric characteristics and CMJ would affect the expected WBGT-induced changes in V_IFT_ performance.

The 30:15_IFT_ was planned four times per season [pre-season (July), *n* = 222; early season (September), *n* = 80, mid-season (January), *n* = 188; end-season (April), *n* = 123]. A total of 642 V_IFT_ records were available from 312 participants who were tested at least once [Median = 2 assessments per player; Min-Max = 1–9] over the course of the two full seasons. During the V_IFT_ testing sessions, temperature ranged from 24.3 to 43.5 °C, whereas relative humidity ranged from 8.0 to 67.0%. Assessments were conducted in different time slots in the afternoon depending on the season, starting at 4 p.m. (December and January; *n* = 94), 5 p.m. (November and February; *n* = 27), 6 p.m. (September, October, March and April; *n* = 65) and 7 p.m. (July, August and May; *n* = 416). Exceptions were made for players who had missed their scheduled session and were tested within one week in the morning between 8 and 9 a.m. (*n* = 42; 6.5% of total observations). Since WBGT was recorded individually for each testing session and linked retrospectively to each V_IFT_ observation, any environmental differences between morning and afternoon/evening sessions were captured within the WBGT variable. These data were derived from regular player monitoring over the course of the season, during which coaching staff and participants received a testing report. The team coaches and players did not receive any input from the research team. Neither anthropometric measurements nor performance tests were conducted during the two Ramadan Fasting periods observed in both seasons.

### 2.2 Participants

312 young football players classified as tier 4 (competing at elite/international level with a training frequency of 4 or 5 sessions per week, depending on weekly fixtures) according to the participant classification framework, took part in this study (23). The players were grouped according to their age as under-14 (U14; *n* = 50), under-15 (U15; *n* = 64), under-16 (U16; *n* = 76), under-17 (U17; *n* = 83), and under-19 (U19; *n* = 88). The 2022/23 and 2023/24 national leagues did not include the U19 and Under-18 (U18), respectively. Thus, to avoid considering single-season data, U18 players from the 2022/23 season were classified as U19. Sixty-six players participated in both seasons. Participants were assessed from the 1st of July 2022 to the 30th of May 2024. As data were derived from routine player monitoring, written informed consent was not required. The requirement for written consent was waived by the Institutional Ethics Committee of Faculty for Human and Business Sciences of Saarland University (Ref No.: 25-11). Players and their guardians were verbally informed upon club registration, and coaching staff and participants received a testing report at the end of each assessment period. A detailed description of the participants' characteristics is reported in Table 1.

**Table 1:** Anthropometric and physical performance characteristics of players from a Middle-East soccer academy.

Variable	Age-group	*n*	Mean ± SD [95% CIs]
Age [years]	U14	109	13.4 ± 0.6 [13.3 to 13.5]
	U15	135	14.2 ± 0.6 [14.1 to 14.3]
	U16	146	15.3 ± 0.6 [15.2 to 15.4]
	U17	122	16.1 ± 0.5 [16.0 to 16.2]
	U19	117	17.0 ± 0.9 [16.9 to 17.2]
Standing height [cm]	U14	109	157.5 ± 9.0 [155.8 to 159.2]
	U15	125	169.7 ± 7.5 [168.4 to 171.0]
	U16	128	172.9 ± 6.6 [171.7 to 174.0]
	U17	104	175.6 ± 5.9 [174.4 to 176.7]
	U19	104	176.5 ± 6.8 [175.2 to 177.8]
Body mass [kg]	U14	114	46.6 ± 8.0 [45.1 to 48.1]
	U15	127	60.5 ± 9.4 [58.9 to 62.2]
	U16	138	63.3 ± 9.7 [61.6 to 64.9]
	U17	108	65.9 ± 8.2 [64.3 to 67.4]
	U19	107	69.3 ± 8.1 [67.8 to 70.9]
Fat mass [%]	U14	85	15.2 ± 5.3 [14.1 to 16.3]
	U15	109	12.9 ± 4.6 [12.1 to 13.8]
	U16	114	11.8 ± 4.3 [11.0 to 12.6]
	U17	85	11.7 ± 2.9 [11.1 to 12.3]
	U19	80	11.0 ± 3.1 [10.3 to 11.7]
CMJ height [cm]	U14	95	30.1 ± 4.9 [29.2 to 31.1]
	U15	103	35.4 ± 4.4 [34.6 to 36.3]
	U16	133	37.6 ± 5.8 [36.6 to 38.6]
	U17	101	39.1 ± 6.3 [37.9 to 40.3]
	U19	72	39.2 ± 5.3 [37.9 to 40.4]
V_IFT_ [km/h]	U14	122	17.6 ± 2.0 [17.2 to 17.9]
	U15	135	18.3 ± 2.0 [17.9 to 18.6]
	U16	145	19.4 ± 2.1 [19.1 to 19.7]
	U17	122	19.6 ± 1.7 [19.3 to 19.9]
	U19	117	19.7 ± 1.8 [19.4 to 20.1]

CMJ, countermovement jump; SD, standard deviation; V_IFT_, latest speed of the 30:15 intermittent fitness test.

### 2.3 Environmental conditions

Environmental conditions, in the form of dry-bulb temperature and WBGT (a heat index that considers air temperature, relative humidity, wind, and solar radiation), were retrospectively linked to all 30-15_IFT_ endurance performance tests. The advantages and disadvantages of using WBGT have been previously described (24–26). The environmental data were obtained from an open-source service (Meteostat.net), which provides hourly meteorological data for specified geographic locations. The service estimates temperature, relative humidity, dew point, wind speed, and air pressure based on a weighted interpolation of up to four weather stations, considering their distances and elevation differences relative to the actual location. Solar radiation was also estimated using the solar angle at the time and location of the test. Finally, the WBGT was estimated based on the method developed by Liljegren et al. (24), which is an acceptable alternative to direct WBGT measurements (14). Data were based on the nearest weather station, located 5.6 km from the study location. Environmental conditions were classified based on the WBGT as “unrestricted” (<22 °C), “low risk” (22 °C–28 °C), “high risk” (28 °C–30 °C) “very high risk” (>30 °C) and “stop play” (WBGT >32 °C), as previously adopted in football research (7, 8).

### 2.4 Testing

#### 2.4.1 Anthropometric measurements

The chronological age on the test date was calculated for each V_IFT_ session by subtracting the players' birth dates from the test dates. Standing height was measured using a portable stadiometer (Seca 213, Seca GmbH, Hamburg, Germany) to the nearest 0.1 cm. Players stood barefoot, upright, with heels together and head in the Frankfurt plane. The measurement was taken at the end of a deep breath, with the headpiece gently lowered to touch the top of the head. All measurements were performed by the same trained assessor to avoid inter-rater error. Body mass and body fat percentage were assessed using bioelectrical impedance analysis (BIA) technology with integrated body composition analysis software (InBody Co., South Korea). Players were measured barefoot and in light clothing, standing upright in a standardized position following the manufacturer's instructions. They were instructed to avoid food and fluid intake for at least two hours before measurement, refrain from intense exercise on the day of assessment, and empty their bladder prior to testing. Standing height, body mass and fat mass were collected once a month over the course of the two seasons and matched to the nearest seasonal V_IFT_. Thus, four out of 10 seasonal anthropometric assessments were considered for analysis.

#### 2.4.2 Countermovement jump

The CMJ performance was assessed using a 1000-Hz dual force plate system (ForceDecks FD Lite, VALD Performance, Brisbane, Australia). CMJ was assessed either on the same day as the 30:15_IFT_, in which case it was always performed before the fitness test, or on a separate day based on the coaches' scheduling, always at the beginning of the training session. To minimize measurement error, only CMJ assessments conducted within a maximum of two weeks before or after the corresponding 30:15_IFT_ session were retained for analysis. After being positioned in an upright stance with their feet shoulder-width apart in the center of the force plates, the players performed a no-arm swing CMJ, placing the arms on the hips, by flexing their knees to a position they perceived to be comfortable. At least a 30-second recovery was allowed between three jump trials. Force-time data derived from the CMJ were uploaded instantaneously to the manufacturer's data administration platform (VALD Hub, VALD Performance, Brisbane, Australia). The final CMJ was retained from the best of three attempts (highest jump height), computed using the impulse-momentum method (27). Each player contributed a median of 2 assessments (Min-Max: 1–9) over the two seasons. Our test-retest reliability was assessed in a subsample of 22 participants using a test-retest approach and was excellent [intraclass correlation coefficient (ICC) ranging from 0.97 to 1.00; unpublished observations].

#### 2.4.3 30:15 intermittent fitness test

Intermittent fitness performance was assessed through the 30-15_IFT_ as described by Buchheit (17). The test consisted of 30-s shuttle runs interspersed with 15-s passive recovery periods. Velocity was set at 8 km·h^−1^ for the first 30-s run and was increased by 0.5 km·h^−1^ every 45-s stage thereafter. Players had to run back and forth between two lines set 40-m apart at a pace governed by a prerecorded audio signal played at appropriate intervals that guided them to adjust their running speed by entering the 3-m zones at each extremity and in the middle of the field while the short beep sounds. During the 15-s recovery period, the subjects walked forward to join the closest line (at the middle or one end of the running area, depending on where the previous run ended) from which they started the next run stage. The aim of the test was to complete as many stages as possible, ending when a player could no longer sustain the imposed running speed or when he was unable to reach the 3-m zone around each line when the audio signal was consecutively sounded 3 times. The speed attained during the last completed stage (V_IFT_) was retained as the intermittent exercise capacity. The results of this test have shown strong associations with directly measured aerobic capacity and excellent reliability in athletes across different team sports (ICC = 0.80–0.99) (16, 21). All tests were conducted on an artificial turf surface. Testing time varied by seasonal period, as described previously. Players were instructed to refrain from strenuous exercise in the 24 h preceding the test and to arrive adequately hydrated. A standardized 6-min warm-up including light dynamic stretching and running technique exercises was completed prior to each testing session.

### 2.5 Statistical analysis

Shapiro–Wilk tests revealed that V_IFT_ within specific age groups was non-normally distributed under unrestricted conditions (*P* < 0.05) but was normally distributed under both low- and high-risk heat stress conditions (*P* > 0.05). Although the original WBGT classification includes a “very high-risk” category (>30 °C), the limited number of observations in this range (WBGT 30 °C–32 °C; U16: *n* = 3, U17: *n* = 3, U19: *n* = 7) precluded its inclusion as a separate category. These cases were instead reclassified into high-risk condition. No observations were recorded under “stop play” conditions (WBGT >32 °C).

To examine whether environmental heat stress was associated with V_IFT_ performance, different linear mixed models were conducted. Environmental stress condition (unrestricted, low risk, and high risk) was treated as a fixed effect, individual subjects as random effects, and V_IFT_ as the dependent variable. The models accounted for unequal participation across players (i.e., varying number of testing sessions) (28). An unstructured covariance structure was applied. The unrestricted condition was used as the reference category to compare observed changes in V_IFT_ under low- and high-risk conditions. Individual characteristics (chronological age, standing height, body mass, body fat percentage, and CMJ height) were entered into the models both separately and simultaneously as covariates. As a sensitivity analysis, seasonal moment (pre-, early-, mid-, end-season) was added as an additional fixed effect to the models including heat stress condition (categorical and continuous WBGT), to evaluate whether the observed heat stress effects on V_IFT_ performance were robust to seasonal variation in fitness. Seasonal moment was not included as an individual covariate with individual characteristics, as it was found to be strongly overlapping with heat stress condition, reflecting the seasonal climate pattern at the study location; its effect was therefore reported separately. The normality of residuals between V_IFT_ and continuous WBGT were checked using histograms and Q-Q plots.

To interpret the magnitude of differences and relationships, an estimate with 95% confidence interval (95% CI) and standardized coefficient (*β*) with 95% CIs, were determined. Significant associations between V_IFT_ and WBGT are presented per 1 °C increase in WBGT. Depending on *β*, effects were categorized into small (*β* = 0.10–0.29), medium (*β* = 0.30–0.49) and large (*β* = >0.50). Repeated-measures correlations and associated 95% CIs were calculated between continuous WBGT and V_IFT_ within each specific group, by modelling the longitudinal dataset as a whole, considering whether the same individual players participated in multiple measurements (29). The magnitudes of correlation were qualitatively interpreted as: trivial (*r* ≤ 0.1), small (*r* = 0.1–0.3), moderate (*r* = 0.3–0.5), large (*r* = 0.5–0.7), very large (*r* = 0.7–0.9) and almost perfect (*r* ≥ 0.9) (30).

Descriptive statistics were reported as estimated marginal means ± 95% CIs unless otherwise described. The significance level was set at *P* < 0.05. Data were analyzed in R Studio (Version 4.3.3) using the packages *performance*, *emmeans*, *lme4*, *rmcorr*, and *lmerTest*.

## 3 Results

### 3.1 Differences in intermittent fitness performance between environmental conditions

A description of V_IFT_ performance values by environmental stress category is reported in Figure 1. Regardless of the age groups, V_IFT_ performance was reduced by ∼0.4 km/h in low-risk conditions and by ∼1.5 km/h in high-risk conditions [*β* = 0.12 (0.03–0.21) and 0.45 (0.37–0.51); *P* < 0.01] compared to unrestricted conditions. When adjusted for single anthropometric characteristics and CMJ performance, those differences were maintained when considering all players or separate age groups. However, when all covariates were entered simultaneously, the differences in V_IFT_ performance during low-risk conditions disappeared for U19 players (lower V_IFT_ by ∼0.23 instead of ∼0.35 km/h; *P* = 0.183). A detailed description of the V_IFT_ estimates under low- and high-stress conditions, compared with unrestricted conditions, is reported in Table 2.

**Figure 1:**
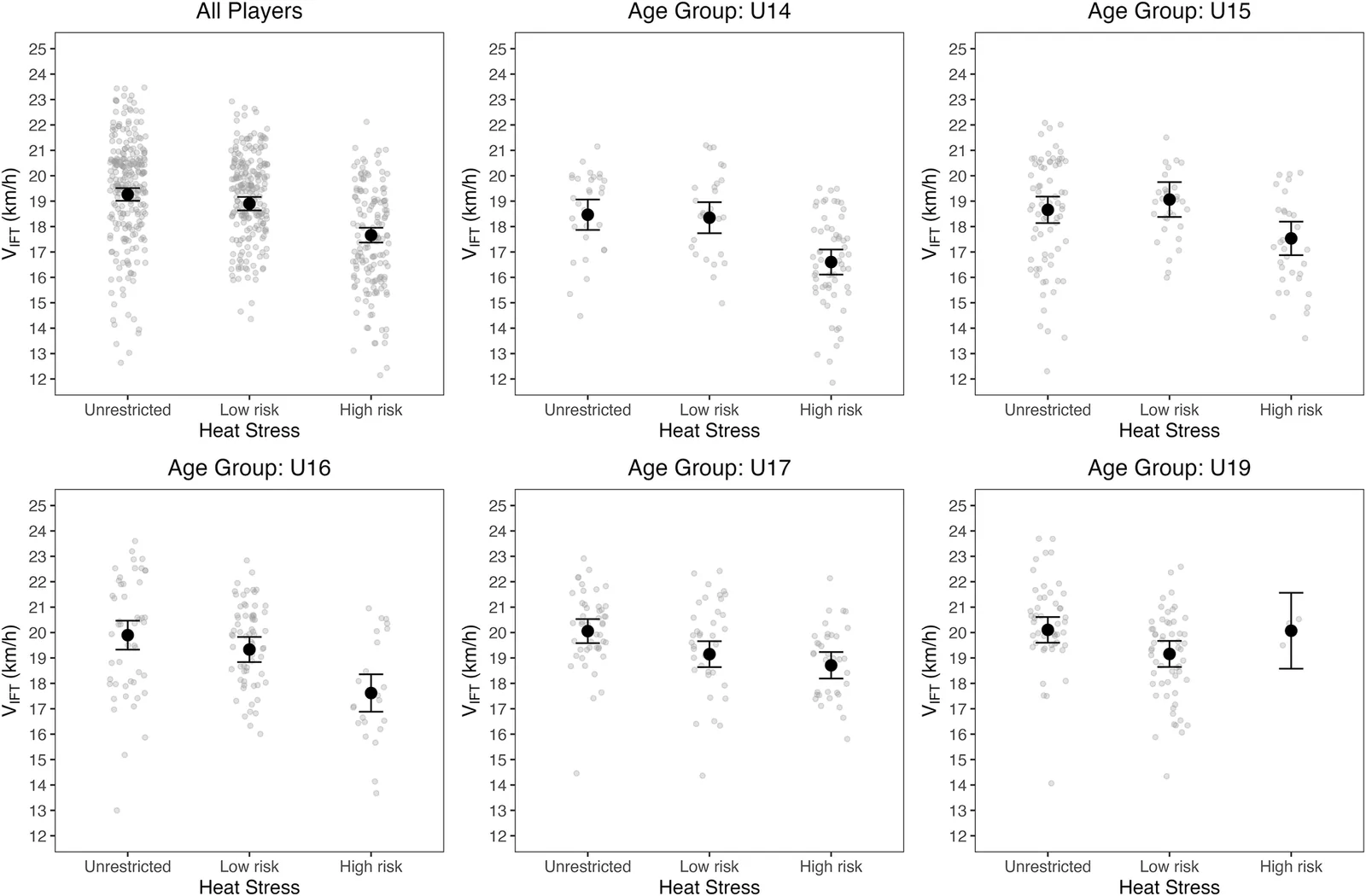
30:15 intermittent fitness performance (V_IFT_) in different environmental condition categories. The black dots are the estimated marginal mean with 95% confidence intervals, whereas the grey dots are the individual data points.

**Table 2:** Differences in 30:15 intermittent fitness test performance (km/h) during low and high risk conditions compared to unrestricted conditions (reference).

Covariate	Age-group	Unrestricted (reference)	Low risk			High risk		
		Estimate [95% CIs]	Estimate	*β* [95% CIs]	*P*	Estimate	*β* [95% CIs]	*P*
No covariate	U14	17.8 [17.4 to 18.2]	−0.1	0.04 [−0.18 to 0.26]	0.721	−1.9	0.55 [0.38 to 0.67]	<.001
	U15	18.4 [17.8 to 19.0]	0.4	0.15 [−0.07 to 0.36]	0.182	−1.1	0.38 [0.18 to 0.55]	<.001
	U16	19.1 [18.7 to 19.5]	−0.5	0.15 [−0.03 to 0.32]	0.090	−2.0	0.43 [0.26 to 0.56]	<.001
	U17	19.3 [18.9 to 19.7]	−0.9	0.34 [0.15 to 0.51]	<.001	−1.3	0.54 [0.36 to 0.69]	<.001
	U19	19.6 [19.2 to 20.0]	−1.0	0.35 [0.15 to 0.52]	<.001	−0.4	0.14 [−0.13 to 0.39]	0.299
	All players	18.6 [18.4 to 18.8]	−0.4	0.12 [0.03 to 0.21]	0.007	−1.5	0.45 [0.37 to 0.51]	<.001
Age	All players	18.6 [18.4 to 18.8]	−0.4	0.14 [0.06 to 0.23]	0.001	−1.4	0.41 [0.33 to 0.49]	<.001
Standing height	U14	17.7 [17.3 to 18.1]	0.0	0.00 [−0.23 to 0.24]	0.973	−1.6	0.50 [0.31 to 0.65]	<.001
	U15	18.1 [17.7 to 18.5]	0.5	0.18 [−0.04 to 0.39]	0.099	−1.0	0.34 [0.14 to 0.52]	0.001
	U16	19.2 [18.8 to 19.6]	−0.5	0.15 [−0.05 to 0.34]	0.128	−2.2	0.48 [0.31 to 0.62]	<.001
	U17	19.6 [19.2 to 20.0]	−0.7	0.30 [0.09 to 0.48]	0.005	−1.5	0.60 [0.42 to 0.73]	<.001
	U19	19.6 [19.2 to 20.0]	−0.9	0.34 [0.13 to 0.52]	0.002	−0.4	0.13 [−0.15 to 0.38]	0.349
	All players	18.6 [18.4 to 18.8]	−0.2	0.09 [−0.01 to 0.18]	0.075	−1.4	0.43 [0.34 to 0.50]	<.001
Body mass	U14	17.9 [17.5 to 18.3]	−0.1	0.02 [−0.20 to 0.25]	0.833	−1.8	0.53 [0.35 to 0.66]	<.001
	U15	18.2 [17.6 to 18.8]	0.5	0.18 [−0.04 to 0.39]	0.107	−1.0	0.35 [0.15 to 0.53]	<.001
	U16	19.2 [18.8 to 19.6]	−0.5	0.14 [−0.05 to 0.31]	0.145	−2.0	0.43 [0.26 to 0.57]	<.001
	U17	19.5 [19.1 to 19.9]	−0.8	0.30 [0.10 to 0.48]	0.004	−1.5	0.60 [0.44 to 0.73]	<.001
	U19	19.7 [19.3 to 20.1]	−0.9	0.34 [0.14 to 0.52]	0.001	−0.4	0.14 [−0.14 to 0.39]	0.312
	All players	18.7 [18.5 to 18.9]	−0.3	0.10 [0.01 to 0.19]	0.036	−1.4	0.43 [0.35 to 0.50]	<.001
Fat mass	U14	18.0 [17.6 to 18.4]	−0.1	0.03 [−0.22 to 0.28]	0.786	−1.5	0.46 [0.25 to 0.63]	<.001
	U15	18.3 [17.9 to 18.7]	0.2	0.06 [−0.17 to 0.28]	0.604	−1.2	0.44 [0.24 to 0.60]	<.001
	U16	19.1 [18.7 to 19.5]	−0.6	0.18 [−0.04 to 0.38]	0.099	−2.2	0.50 [0.31 to 0.64]	<.001
	U17	19.7 [19.3 to 20.1]	−0.7	0.28 [0.04 to 0.48]	0.019	−1.6	0.64 [0.46 to 0.77]	<.001
	U19	19.6 [19.2 to 20.0]	−0.9	0.36 [0.12 to 0.56]	0.003	−0.3	0.09 [−0.20 to 0.36]	0.536
	All players	18.7 [18.5 to 18.9]	−0.3	0.12 [0.02 to 0.22]	0.016	−1.5	0.45 [0.37 to 0.53]	<.001
CMJ height	U14	17.9 [17.5 to 18.3]	−0.1	0.04 [−0.20 to 0.27]	0.759	−1.6	0.53 [0.34 to 0.68]	<.001
	U15	18.4 [18.0 to 18.8]	0.3	0.09 [−0.14 to 0.30]	0.443	−1.2	0.38 [0.17 to 0.56]	<.001
	U16	19.2 [18.8 to 19.6]	−0.4	0.11 [−0.07 to 0.29]	0.220	−1.9	0.41 [0.23 to 0.55]	<.001
	U17	19.7 [19.3 to 20.1]	−0.6	0.23 [0.01 to 0.43]	0.035	−1.5	0.61 [0.44 to 0.74]	<.001
	U19	19.7 [19.1 to 20.3]	−0.8	0.27 [0.00 to 0.51]	0.044	−0.5	0.17 [−0.17 to 0.47]	0.311
	All players	18.9 [18.7 to 19.1]	−0.3	0.11 [0.01 to 0.21]	0.027	−1.5	0.44 [0.36 to 0.52]	<.001
All covariates	U14	18.0 [17.4 to 18.6]	−0.1	0.04 [−0.24 to 0.31]	0.795	−1.5	0.50 [0.26 to 0.68]	<.001
	U15	18.2 [17.8 to 18.6]	0.0	0.01 [−0.23 to 0.25]	0.946	−1.2	0.42 [0.19 to 0.60]	<.001
	U16	19.3 [18.7 to 19.9]	−0.6	0.17 [−0.04 to 0.38]	0.109	−2.0	0.47 [0.27 to 0.62]	<.001
	U17	19.7 [19.3 to 20.1]	−0.7	0.30 [0.05 to 0.51]	0.019	−1.6	0.64 [0.45 to 0.78]	<.001
	U19	19.4 [18.6 to 20.2]	−0.5	0.23 [−0.12 to 0.52]	0.183	−0.3	0.12 [−0.24 to 0.45]	0.497
	All players	18.7 [18.5 to 18.9]	−0.4	0.13 [0.03 to 0.24]	0.015	−1.4	0.45 [0.36 to 0.53]	<.001

Estimates are expressed in km/h.

CI, confidence interval; CMJ, countermovement jump.

### 3.2 Relationship between wet-bulb globe temperature and intermittent fitness performance

WBGT was negatively associated with V_IFT_ performance (*P* < 0.05). The magnitude of correlations was small in U19 [*r* (95% CIs) = −0.28 (−0.52 to −0.01); *P* = 0.015], moderate in U14, U15 and U16 (*r* = −0.50 to −0.31; *P* < 0.05) and large in U17 [*r* (95% CIs) = −0.61 (−0.74 to −0.43); *P* < 0.001]. A representation of the correlation between WBGT and V_IFT_ is reported in Figure 2.

**Figure 2:**
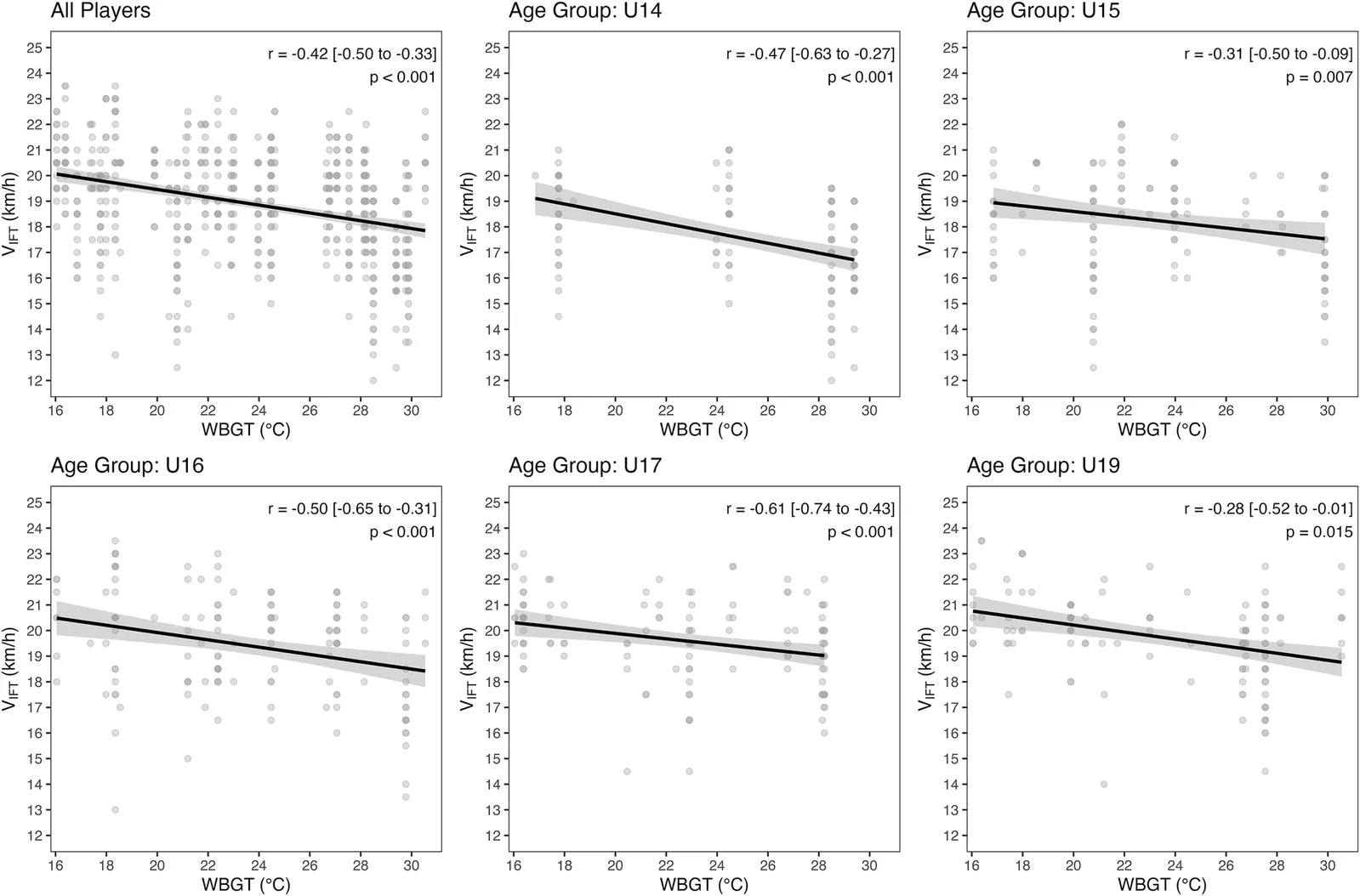
Within-subject correlations between continuous wet-bulb globe temperature (WBGT) and last velocity of the 30:15 intermittent fitness test (V_IFT_).

For every increase in 1 °C in WBGT, V_IFT_ performance was reduced by 0.09 to 0.15 km/h depending on the age-group (*r* = −0.48 to −0.30; *P* < 0.01). When adjusted to single anthropometric characteristics and CMJ performance, those relationships were maintained in all age-groups (*P* < 0.05). When all covariates were entered simultaneously, the relationship disappeared for the U19 age-group (*P* = 0.205). A detailed description of the relationship between WBGT and V_IFT_ performance is presented in Table 3.

**Table 3:** Relationship between wet bulb globe temperature and 30:15 intermittent fitness test performance.

Covariate	Age-group	Estimate [95% CIs]	*P*	*β* [95%CIs]
No covariates	U14	−0.15 [−0.21 to −0.10]	<0.001	−0.48 [−0.66 to −0.29]
	U15	−0.10 [−0.15 to −0.04]	0.001	−0.30 [−0.48 to −0.12]
	U16	−0.16 [−0.23 to −0.09]	<0.001	−0.41 [−0.58 to −0.24]
	U17	−0.12 [−0.16 to −0.08]	<0.001	−0.46 [−0.61 to −0.30]
	U19	−0.09 [−0.15 to −0.04]	0.001	−0.45 [−0.70 to −0.20]
	All players	−0.13 [−0.15 to −0.10]	<0.001	−0.42 [−0.50 to −0.34]
Age	All players	−0.12 [−0.14 to −0.09]	<0.001	−0.41 [−0.49 to −0.33]
Standing height	U14	−0.15 [−0.21 to −0.09]	<0.001	−0.44 [−0.63 to −0.26]
	U15	−0.08 [−0.14 to −0.02]	0.006	−0.26 [−0.44 to −0.07]
	U16	−0.17 [−0.24 to −0.10]	<0.001	−0.44 [−0.62 to −0.26]
	U17	−0.12 [−0.16 to −0.08]	<0.001	−0.51 [−0.68 to −0.35]
	U19	−0.09 [−0.14 to −0.03]	0.003	−0.40 [−0.66 to −0.15]
	All players	−0.12 [−0.15 to −0.10]	<0.001	−0.41 [−0.49 to −0.32]
Body mass	U14	−0.16 [−0.21 to −0.10]	<0.001	−0.48 [−0.66 to −0.30]
	U15	−0.09 [−0.14 to −0.03]	0.004	−0.27 [−0.45 to −0.09]
	U16	−0.17 [−0.24 to −0.10]	<0.001	−0.43 [−0.60 to −0.26]
	U17	−0.12 [−0.16 to −0.09]	<0.001	−0.52 [−0.68 to −0.36]
	U19	−0.09 [−0.14 to −0.04]	0.002	−0.42 [−0.67 to −0.16]
	All players	−0.12 [−0.15 to −0.10]	<0.001	−0.42 [−0.50 to −0.33]
Fat mass	U14	−0.14 [−0.20 to −0.08]	<0.001	−0.42 [−0.62 to −0.22]
	U15	−0.10 [−0.15 to −0.04]	0.001	−0.33 [−0.52 to −0.14]
	U16	−0.18 [−0.25 to −0.11]	<0.001	−0.45 [−0.62 to −0.27]
	U17	−0.13 [−0.17 to −0.09]	<0.001	−0.54 [−0.71 to −0.37]
	U19	−0.06 [−0.12 to −0.01]	0.036	−0.28 [−0.54 to −0.02]
	All players	−0.13 [−0.15 to −0.10]	<0.001	−0.41 [−0.50 to −0.33]
CMJ height	U14	−0.14 [−0.20 to −0.09]	<0.001	−0.45 [−0.63 to −0.27]
	U15	−0.10 [−0.17 to −0.04]	0.003	−0.30 [−0.50 to −0.11]
	U16	−0.15 [−0.22 to −0.08]	<0.001	−0.38 [−0.56 to −0.21]
	U17	−0.12 [−0.16 to −0.09]	<0.001	−0.53 [−0.70 to −0.36]
	U19	−0.08 [−0.14 to −0.02]	0.018	−0.38 [−0.69 to −0.07]
	All players	−0.13 [−0.15 to −0.10]	<0.001	−0.42 [−0.50 to −0.33]
All covariates	U14	−0.10 [−0.17 to −0.02]	0.015	−0.29 [−0.52 to −0.06]
	U15	−0.10 [−0.16 to −0.03]	0.006	−0.30 [−0.51 to −0.09]
	U16	−0.16 [−0.23 to −0.08]	<0.001	−0.39 [−0.57 to −0.21]
	U17	−0.12 [−0.16 to −0.08]	<0.001	−0.52 [−0.70 to −0.34]
	U19	−0.04 [−0.10 to 0.02]	0.205	−0.18 [−0.48 to 0.11]
	All players	−0.12 [−0.15 to −0.10]	<0.001	−0.40 [−0.49 to −0.31]

Estimates ± standard error (SE) are expressed in km/h.

CI, confidence interval; CMJ, countermovement jump.

### 3.3 Association between seasonal moment, environmental conditions and intermittent fitness performance

The distribution of heat stress conditions varied across seasonal moments: 83.1% of pre-season testing sessions occurred under high-risk conditions, whereas 0.5% of mid-season sessions. Despite, the reduction in V_IFT_ performance during high-risk conditions remained significant after adjusting for seasonal moment for all players (−1.01 km/h vs. −1.54 km/h unadjusted; *P* < 0.001). Seasonal moment was itself significantly associated with V_IFT_ performance (*P* < 0.001), with the lowest values observed in pre-season and the highest in mid-season. Similarly, the negative relationship between WBGT and V_IFT_ performance remained significant for all players after adjustment for seasonal moment (−0.095 km/h per 1 °C vs. −0.132 km/h per 1 °C unadjusted; *P* < 0.001).

## 4 Discussion

The key finding of this study is that the intermittent running performance of highly trained youth football players, assessed using the 30:15 IFT, was negatively affected by environmental heat stress, independent of anthropometric characteristics and CMJ. The performance decrement ranged from ∼0.09 to 0.15 km/h for every 1 °C increase in WBGT. The observed heat-induced performance decrements likely stem from multiple interrelated physiological mechanisms. Elevated environmental temperature substantially increases core and muscle temperatures, and these changes may impair neuromuscular function and reduce the capacity for high-intensity intermittent exercise (31). Heat stress also increases cardiovascular strain by redirecting blood flow to the skin for thermoregulation, thereby reducing oxygen delivery to working muscles and decreasing maximal aerobic capacity (6). Additionally, the 30:15IFT requires repeated high-intensity accelerations and decelerations, which generate substantial metabolic heat production that compounds the thermal stress imposed by environmental conditions. Youth athletes may be particularly vulnerable to these effects due to their lower sweating capacity, higher surface-area-to-mass ratio, and less mature cardiovascular responses compared to adults (9). The finding that U19 players showed attenuated heat effects in low-risk conditions suggests that age-related improvements in thermoregulatory capacity and/or heat acclimatization may partially offset environmental thermal stress, although this warrants further investigation.

Compared to unrestricted thermal conditions (<22 °C WBGT), V_IFT_ performance decreased by ∼0.4 km/h under low-risk conditions (22 °C–28 °C WBGT) and by ∼1.5 km/h under high-risk conditions (28 °C–30 °C WBGT). This is consistent with international recommendations for safe sport participation in the heat issued by the International Olympic Committee (13), suggesting that the performance decrements observed in the present study become practically meaningful precisely at the environmental boundaries beyond which governing bodies advise caution. These results remained significant even after adjusting for anthropometric and neuromuscular function. Previous studies have shown that endurance capacity (32) and repeated sprints performance (31) are lower in the heat compared to thermo-neutral environment conditions (31). The linear relationship between WBGT and VIFT (∼0.09–0.15 km/h reduction per 1 °C-WBGT increase) supports the argument that higher heat loads impair performance, particularly in younger age-groups. The strength of the WBGT-performance association varied across age-groups, with U17 players showing the highest correlation (*r* = −0.61). This could be because younger athletes (∼16 to 17 years old) have lower sweat rates, higher core temperatures, and reduced cardiovascular stability in the heat, leading to diminished tolerance and greater performance decrements (6). In contrast, U19 players showed minimal performance reduction in low-risk conditions after adjustment for all covariates, potentially reflecting greater age-related improvements in thermoregulatory capacity or accumulated heat acclimatization resulting from prolonged exposure to hot training environments over multiple seasons. When adjusted for individual anthropometric and neuromuscular characteristics (e.g., height, body mass, fat mass, and CMJ), differences in V_IFT_ performance under heat stress remained consistent across age-groups. However, when all covariates were entered simultaneously, the performance decrement under low-risk conditions disappeared in U19 players (∼0.23 vs. ∼0.35 km/h). This suggests that older adolescents might be able to better tolerate environmental stress. Such findings align with prior literature indicating enhanced thermoregulatory efficiency, cardiovascular stability, and accumulated heat exposure experience in late-adolescent players (17–19 years) (6, 9) highlighting the importance of interpreting environmental effects in light of player age and physical development.

Our results are particularly relevant given that the 30:15_IFT_ is often administered outdoors in youth football contexts. While the test is known for its high reliability and training sensitivity (17), our findings highlight that environmental conditions can substantially bias test outcomes. This is especially important in hot and humid regions, where test results may underestimate a player's actual aerobic capacity. As noted by Asimakidis et al. (15), the 30:15_IFT_ is the most widely used intermittent fitness test among football practitioners, making environmental control or adjustment critical when interpreting performance. The present data aligns with growing evidence supporting the implementation of heat mitigation strategies in football environments, emphasizing the urgent need for better mitigation strategies and environmental monitoring during training and testing, especially for youth athletes (4, 5).

The findings of the present study can also help in individualizing training based on 30:15_IFT_. As an example, if a player's V_IFT_ was 18 km/h, the strength and conditioning coach can use this value to estimate the maximal oxygen uptake based on the 80%–85% of the VIFT, which in this case would be 14.4–15.3 km/h. However, when the 30:15 IFT is conducted at elevated temperatures, adjustments to the final speed might be needed to prescribe endurance training under unrestricted conditions. Depending on how much higher the WBGT on the test day was compared to the training day, V_IFT_ may be estimated to increase by approximately one running stage (0.5 km/h) for every ∼5 °C increase in WBGT. In this case, the player's test score can be corrected from 18 to 18.5 or 19 km/h, resulting in a higher maximal oxygen uptake (14.8–16.1 km/h instead of 14.4–15.3 km/h) estimated for unrestricted environmental conditions, and prescribe endurance training accordingly.

This study is not exempt from limitations. The major criticism is the lack of control over the varying times at which testing took place throughout the year. Indeed, performance fluctuations are expected throughout the year due to detraining (testing after summer break and/or long breaks) and training. Additionally, biological maturation (e.g., peak height velocity) was not assessed, despite its known influence on both performance and heat tolerance (6, 33). Internal physiological responses during the 30:15_IFT_, such as heart rate, rating of perceived exertion, or thermal comfort, were not collected. In this context, submaximal heart rate responses to the 30:15_IFT_ based on the first 4- and 6-min of the test (included as a warm-up protocol) have shown good reliability in professional football players (34). Including such measures would provide insight into the mechanisms (e.g., cardiovascular strain, perceived exertion) underlying heat-induced performance decline. Future studies might consider standardized testing windows or incorporate training load data for more precise modeling. Body composition measurements were conducted in the afternoon and in a non-fasted state, which may have introduced variability due to recent food or fluid intake. Non-standardized measurement timing can affect hydration status and gastrointestinal content, potentially leading to overestimation of body mass and fat mass, particularly when using field-based methods. The absence of individual data on players' hydration status, prior heat acclimatization, sleep quality, and biological maturation represents a notable limitation, as each of these factors may independently influence the observed performance responses to heat stress (35, 36). Importantly, WBGT values were estimated using meteorological data from a weather station located 5.6 km from the test site, rather than measured *in situ* during testing sessions. While this approach has been validated against direct measurements (14), it may not fully capture the microclimate conditions experienced by players during testing, particularly variations in solar radiation, wind patterns, or surface heat. Future studies should incorporate on-site WBGT monitoring to improve measurement precision. Finally, V_IFT_ performance varied significantly across seasonal periods, independent of heat-stress conditions. Reassuringly, the heat stress effect on V_IFT_ remained significant in both magnitude and direction after adjusting for seasonal moment, indicating that our main findings are not solely attributable to seasonal variation in players' fitness. However, given that heat stress conditions in our dataset were closely tied to the time of year, with high-risk conditions concentrated in pre-season and unrestricted conditions predominantly observed in mid-season, it was not possible to fully separate the independent contributions of environmental heat stress and seasonal variation in fitness to the observed performance changes. Future studies conducted across more climatically homogeneous regions, or using experimentally controlled heat exposure, would help isolate these effects more clearly.

In summary, this study provides novel evidence that both individual characteristics and environmental heat stress significantly affect performance in the 30:15_IFT_ among highly trained youth football players. Environmental heat stress was associated with clear reductions in V_IFT_ performance, independently of anthropometric characteristics and CMJ. The performance decrement ranged from ∼0.09 to 0.15 km/h for every 1 °C increase in WBGT. These findings emphasize that field testing under hot conditions can lead to underestimated fitness outcomes, particularly in younger athletes who are more vulnerable to heat due to their less developed thermoregulatory systems (6). As the 30:15_IFT_ is increasingly used for training prescription and performance monitoring in youth football (15), these findings reinforce the importance of contextualizing test results by both individual developmental status and environmental conditions.

## 5 Conclusions

Our findings provide observations for practitioners using the 30:15IFT to profile young football players and prescribe training intensities. Based on the observed relationship between WBGT and V_IFT_ performance (∼0.5 km/h decline per 5 °C WBGT increase), we recommend standardizing testing conditions whenever possible by conducting assessments during cooler periods (WBGT <22 °C) to minimize environmental confounding and ensure reliability. When testing in heat is unavoidable, practitioners may adjust V_IFT_ results by adding approximately +0.5 km/h for low-risk conditions (WBGT 22 °C–28 °C) or +1.5 km/h for high-risk conditions (WBGT ≥28 °C) to facilitate longitudinal comparisons and approximate the performance expected under unrestricted conditions. Similarly, when prescribing training intensities based on V_IFT_ obtained in heat, coaches should reduce target velocities by the same magnitude to avoid overloading players during hot training sessions. We recommend using accessible WBGT estimation tools (e.g., Meteostat.net, weather station data) to document thermal conditions during both testing and training, enabling more informed decision-making regarding player assessment and load management. Finally, practitioners should consider age-specific responses, as younger players (U14-U17) showed consistent heat-related performance decrements while U19 players demonstrated greater resilience in low-risk conditions, possibly reflecting thermoregulatory maturation. Given the observational nature of this study, the proposed estimates are based on observed associations and should be interpreted cautiously, as they do not imply causal relationships between heat stress and performance decrements. Future prospective studies incorporating direct on-site WBGT measurements and physiological monitoring are needed to confirm and refine these recommendations.

## Data Availability

Data cannot be shared publicly because it contains potentially sensitive information and was obtained from a third party (i.e., Football Academy, Al Ain Sports & Cultural Club), and access restrictions apply. Data are available for researchers who meet the criteria for access to confidential data. Requests to access the datasets should be directed to Vincenzo Rago, vincenzorago24@gmail.com.
